# Resistance to Antimicrobials Mediated by Efflux Pumps in *Staphylococcus aureus*

**DOI:** 10.3390/antibiotics2010083

**Published:** 2013-03-13

**Authors:** Sofia S. Costa, Elisabete Junqueira, Cláudia Palma, Miguel Viveiros, José Melo-Cristino, Leonard Amaral, Isabel Couto

**Affiliations:** 1 Grupo de Micobactérias, Unidade de Microbiologia Médica, Instituto de Higiene e Medicina Tropical, Universidade Nova de Lisboa (IHMT, UNL), 1349-008 Lisbon, Portugal; E-Mails: scosta@ihmt.unl.pt (S.S.C.); ejunqueira@ihmt.unl.pt (E.J.); cpalma@ihmt.unl.pt (C.P.); MViveiros@ihmt.unl.pt (M.V.); 2 Centro de Recursos Microbiológicos (CREM), UNL, 2829-516 Caparica, Portugal; 3 Centro Hospitalar Lisboa Norte E.P.E., Instituto de Microbiologia, Instituto de Medicina Molecular, Faculdade de Medicina, Universidade de Lisboa, 1649-028 Lisbon, Portugal; E-Mail: melo_cristino@fm.ul.pt; 4 Grupo de Medicina Tropical e do Viajante, Centro de Malária e Doenças Tropicais (CMDT), IHMT, UNL, 1349-008 Lisbon, Portugal; E-Mail: LAmaral@ihmt.unl.pt

**Keywords:** *Staphylococcus aureus*, resistance, antibiotics, biocides, efflux

## Abstract

Resistance mediated by efflux has been recognized in *Staphylococcus aureus* in the last few decades, although its clinical relevance has only been recognized recently. The existence of only a few studies on the individual and overall contribution of efflux to resistance phenotypes associated with the need of well-established methods to assess efflux activity in clinical isolates contributes greatly to the lack of solid knowledge of this mechanism in *S. aureus*. This study aims to provide information on approaches useful to the assessment and characterization of efflux activity, as well as contributing to our understanding of the role of efflux to phenotypes of antibiotic resistance and biocide tolerance in *S. aureus* clinical isolates. The results described show that efflux is an important contributor to fluoroquinolone resistance in *S. aureus* and suggest it as a major mechanism in the early stages of resistance development. We also show that efflux plays an important role on the reduced susceptibility to biocides in *S. aureus*, strengthening the importance of this long neglected resistance mechanism to the persistence and proliferation of antibiotic/biocide-resistant *S. aureus* in the hospital environment.

## 1. Introduction

Efflux pumps are membrane proteins that have the function of detoxifying cells by expelling noxious molecules [1]. The extrusion of antimicrobial compounds, such as antibiotics and biocides, is considered to be an “accidental function” of such efflux systems [2,3]. Nevertheless, efflux-mediated resistance towards antimicrobial compounds is increasingly recognized as an important resistance mechanism in bacteria [4]. Efflux pumps present different substrate specificities; some are specific to an antibiotic or a class of antibiotics, whereas multidrug efflux pumps, as the name implies, have the capacity to extrude more than one class of antibiotics and/or other antimicrobial compounds [5]. These latter efflux systems are of foremost relevance, since they can bestow the bacterial cell with a phenotype of resistance to multiple drugs in addition to promoting cross-resistance between antibiotics and other antimicrobial compounds usually used to prevent and control healthcare associated infections [4].

In *Staphylococcus aureus*, several specific efflux pumps have been associated with resistance to antibiotics, such as tetracycline (Tet(K), Tet(L)) and macrolides (Mef(A), Msr(A)) [5]. Also, several multidrug efflux pumps have been described that are associated with resistance to antibiotics (e.g., fluoroquinolones) and to biocides, such as NorA, NorB, NorC, MepA and MdeA [5]. Other multidrug efflux pumps expel only biocides, as is the case of QacA/B and Smr [5]. In general, specific efflux pumps can be found either in the chromosome or in plasmids, while multidrug efflux pumps are mainly located in the chromosome, with the exception of QacA/B and Smr, which have only been described in plasmids [5].

Despite the increasing number of *S. aureus* efflux pumps identified with the potential to contribute to the resistance towards clinically relevant antibiotics and other antimicrobial compounds, few studies have been undertaken to ascertain the collective and individual contribution of efflux systems to resistance phenotypes in clinical isolates [6,7,8], resulting in little information being available. One of the underlying reasons for this is the lack of established methods to assess efflux activity, mainly in the definition of threshold values for the attribution of “basal” *versus* “increased” levels for efflux activity, as well as the determination of the role of each pump on the overall efflux activity. Several approaches have been used to identify active efflux systems in bacteria, such as the use of radiolabelled substrates, fluorometric assays or the determination of the minimum inhibitory concentration (MIC) for different substrates in the presence of efflux inhibitors (EIs) [7,9,10]. In our group, we have developed methods based on ethidium bromide (EtBr), a substrate of the majority of the *S. aureus* multidrug efflux pumps, that has been proven reliable for the assessment of efflux activity in bacteria [11], namely the EtBr-agar cartwheel method [12], that allows the screening of large collections of clinical isolates to detect isolates with increased efflux activity. The monitoring of EtBr efflux in clinical isolates by real-time fluorometry [13] permits a more extensive characterization of that efflux activity and can be used to confirm, on a real-time basis, the results of the EtBr-agar cartwheel method. The use of EtBr has also been proven useful by other groups in detecting isolates with increased efflux activity [14]. The information obtained from these methods can then be complemented by the determination of MICs of effluxable substrates in the presence of efflux inhibitors. 

This work describes the use of these approaches to study the role played by efflux on the resistance to antimicrobial agents, including antibiotics and biocides, on a collection of *S. aureus* strains of clinical origin and how this efflux activity may contribute to the persistence of *S. aureus* cells over-expressing efflux pumps on the clinical environment.

## 2. Results and Discussion

### 2.1. Evaluation of Efflux Activity

**Figure 1:**
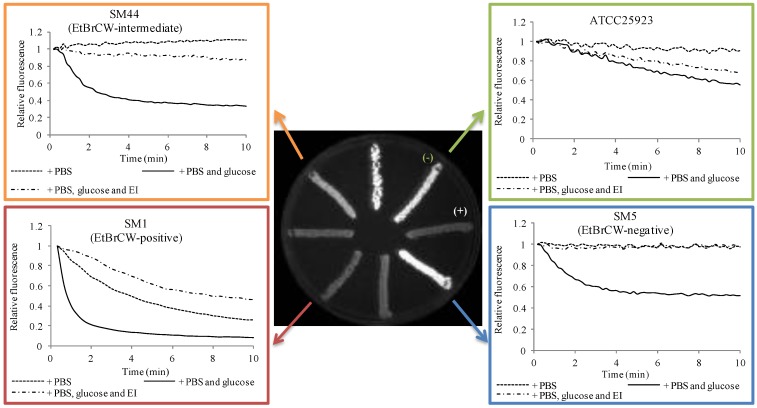
Characterization of reference and clinical isolates according to their efflux capacity. In green: reference strain *S. aureus* ATCC25923; in blue: EtBr-agar cartwheel (EtBrCW)-negative isolate SM5; in orange: EtBrCW-intermediate isolate SM44; in red: EtBrCW-positive isolate SM1. Central figure: screening of efflux activity by the EtBrCW method. Trypticase soy agar (TSA) plate supplemented with 2.5 mg/L EtBr streaked with representative isolates. (−) and (+): strains ATCC25923 and ATCC25923_EtBr_, used respectively as negative and positive controls for EtBr efflux. Graphics: evaluation of efflux activity by real-time fluorometry. Efflux assays for representative isolates are shown for cells resuspended in Phosphate buffered saline (PBS), in PBS plus glucose (0.4%) or PBS plus glucose and the efflux inhibitor, verapamil (at the sub-inhibitory concentration of 200 mg/L). The data presented was normalized against the data obtained in conditions of no efflux (absence of glucose and presence of 200 mg/L of verapamil).

The EtBr-agar cartwheel (EtBrCW) method is a practical methodology to assess the presence of increased efflux activity in large collections of clinical isolates of different bacterial species [12]. This method allows the comparison of different isolates on the basis of their capacity to extrude EtBr. The isolates are streaked in solid media containing increasing concentrations of EtBr and the fluorescence emitted, which is inversely proportional to their capacity to extrude the compound, is compared to the fluorescence of control strains. Using this approach to test a collection of 52 ciprofloxacin-resistant *S. aureus*, we could discriminate these isolates in three distinct groups: a group of twelve isolates that showed fluorescence only at the highest EtBr concentration tested, presumably with increased efflux activity and designated EtBrCW-positive; a group of thirty-three isolates that showed fluorescence at the lowest EtBr concentrations tested and denominated EtBrCW-negative; and a third group of seven isolates showing fluorescence at intermediate concentrations of EtBr and denominated EtBrCW-intermediate isolates [7] (Figure 1).

Further characterization of these isolates by a fluorometric assay that detects EtBr efflux by assessing in real-time the loss of EtBr fluorescence in bacterial cells previously loaded with this dye enabled us to corroborate the preliminary characterization of the isolates by the EtBrCW method (Figure 1). In particular, the increased efflux activity present in EtBrCW-positive isolates (Figure 1, red) is demonstrated by the lack of fluorescence in EtBr-agar plates together with a prompt EtBr efflux by real-time fluorometry, whereas EtBrCW-negative isolates (Figure 1, blue) emit a strong fluorescence in EtBr-agar plates and show only slight EtBr efflux. On the other hand, EtBrCW-intermediate isolates show intermediate fluorescence and EtBr efflux (Figure 1, orange). This analysis also showed that basal efflux activity is always present in *S. aureus*, as shown by the reference strain. ATCC25923 (Figure 1, green). 

Altogether, these two EtBr-based assays proved to be valuable tools to screen for increased efflux activity in clinical isolates of *S. aureus*, making it possible to differentiate strains with varying levels of efflux activity. As stated previously, EtBr is a common substrate of multidrug efflux pumps, which can also extrude other antimicrobial compounds, such as the antibiotics fluoroquinolones and biocides and, thus, used as a screening marker for efflux, leading to resistance towards fluoroquinolones and biocides. 

### 2.2. Contribution of Efflux to Fluoroquinolone Resistance

Fluoroquinolones are a class of antibiotics that possess a broad spectra of activity, including methicillin-resistant *S. aureus* (MRSA) [15]. However, the swift development of resistance to these antibiotics has impaired their clinical relevance [16]. At present, in Europe, around 25% of *S. aureus* isolates are resistant to fluoroquinolones, a percentage that increases to 90% among MRSA isolates [17].

Resistance to fluoroquinolones in *S. aureus* is usually associated with the occurrence of mutations in the target genes, *grlA/B* and *gyrA/B*, that code for Topoisomerase IV (GrlA/B) and DNA gyrase (GyrA/B) proteins, respectively [16]. These mutations usually occur in a precise region denominated quinolone resistance-determining region (QRDR) and generate proteins with lower affinity for fluoroquinolones [16]. Several studies have shown that Topoisomerase IV is the primary target of fluoroquinolones in *S. aureus*. Accordingly, *in vitro* studies have demonstrated that emergence of fluoroquinolone resistance is associated with acquisition of mutations first in the *grlA* gene followed by mutations in the *gyrA* gene [18]. Also, fluoroquinolone resistant clinical isolates with mutations only in the *gyrA* gene are uncommon [19]. Moreover, quinolone resistant isolates with a single or double mutation in the *grlA* gene present high-level resistance when a *gyrA* mutation is acquired [19,20]. The occurrence of mutations in the *grlB* and *gyrB* genes has been shown to be infrequent [16]. Altogether, mutations in the QRDR of these genes are linked to high-level resistance to fluoroquinolones in *S. aureus* clinical isolates [16].

Resistance to fluoroquinolones mediated by efflux has been described in *S. aureus* clinical isolates for the last two decades [9,18,21,22,23,24,25,26,27,28]. However, these studies related only to the role of the NorA efflux pump. Nowadays, it is known that at least three other multidrug efflux pumps, namely NorB, NorC and MepA, have been described as having fluoroquinolones as a substrate [29,30,31], although their actual contribution to clinical fluoroquinolone resistance remains uncertain. A few studies have been conducted with clinical isolates where an association between fluoroquinolone resistance and these efflux systems was explored [6,7,8]. 

**Figure 2:**
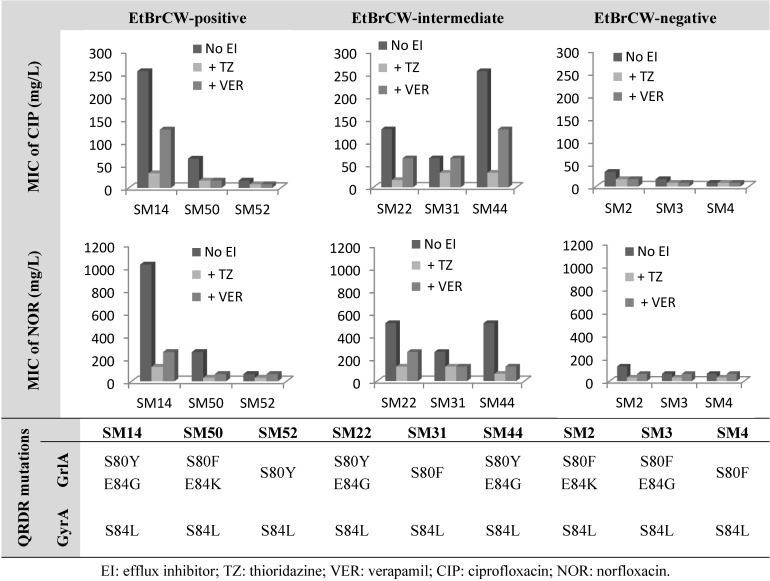
Effect of the efflux inhibitors thioridazine (TZ) and verapamil (VER), at subinhibitory concentrations (12.5 mg/L and 200 mg/L, respectively), on the minimum inhibitory concentration (MIC) values of ciprofloxacin and norfloxacin for representative isolates of the EtBrCW-positive, EtBrCW-intermediate and EtBrCW-negative groups, each carrying different mutations conferring fluoroquinolone resistance (data for EtBrCW-positive and -negative strains from our previous study, [7]).

The effect of the known efflux inhibitors, thioridazine (TZ) and verapamil (VER) [7], on the MIC levels of fluoroquinolones was evaluated, and mutations conferring fluoroquinolone resistance were screened. Reserpine is usually used to assess efflux activity in *S. aureus*, but it was not tested in this study, since previous data by our group has revealed that this compound has a mild inhibitory activity [7]. The isolates presented in Figure 2 are representative of each of the groups established previously, according to efflux capacity. Detailed data on MIC values is provided in Supplementary Table S1.

The data presented in Figure 2 shows that independently of the mutations carried by each strain in both *grlA* and *gyrA* genes, which alter the target affinity of these antibiotics, efflux is an important component of the resistance level. This can be observed in the effect of the efflux inhibitor, TZ, on the MICs of the two fluoroquinolones tested for these isolates. The inhibitory effect of TZ was shown to be higher for strains with higher efflux activity, namely the ones classified as EtBrCW-positive or EtBrCW-intermediate, for which a two- to eight-fold reduction in the MICs of the two fluoroquinolones was observed, whereas this reduction was only two-fold for the EtBrCW-negative isolates. Verapamil showed a weaker effect, resulting in MIC reductions that ranged from none to four-fold for EtBrCW-positive and -intermediate isolates and none to two-fold for EtBrCW-negative isolates. Comparing the data gathered for the EtBrCW-intermediate isolates with the data of the remaining isolates, it could be observed that EtBrCW-intermediate isolates are more similar to EtBrCW-positive isolates. All these isolates carried mutations in both *grlA* and *gyrA* genes that have been described in the literature as being involved in high-level resistance to fluoroquinolones [22,32]. Accordingly, our isolates present ciprofloxacin MICs that range between 16 to 256 mg/L for EtBrCW-positive and -intermediate isolates and 8 to 32 mg/L for EtBrCW-negative isolates; whereas the norfloxacin MICs vary between 64 to 1,024 mg/L for EtBrCW-positive and -intermediate isolates, and 64 to 128 mg/L for EtBrCW-negative isolates. However, part of this resistance can be attributable to efflux, as seen in the degree of MIC reductions by the efflux inhibitors tested in Figure 2. We also observed that although isolates carrying a double mutation in *grlA* and a single mutation in *gyrA* presented higher MICs, as described in the literature, they also suffered higher MIC reductions with the efflux inhibitors, particularly with TZ (two- to eight-fold) in comparison with isolates carrying a single mutation in both genes (none to two-fold). Regardless of the type of combination of QRDR mutations presented by the isolates, the presence of TZ reduced the MICs of ciprofloxacin to 8–32 mg/L and the MICs of norfloxacin to 32–128 mg/L (Figure 2 and Supplementary Table S1). However, the fluoroquinolone resistance phenotype was not fully reverted by TZ, since all isolates remained resistant to either ciprofloxacin or norfloxacin in the presence of the inhibitor, as expected, due to the presence of mutations. 

These results reveal that efflux is an important contributor to fluoroquinolone resistance in *S. aureus.* While mutations in the QRDR of *grlA* and *gyrA* genes confer resistance up to a certain level, in particular 8 to 32 mg/L for ciprofloxacin and 32 to 128 mg/L for norfloxacin, the remaining resistance may be attributable to efflux, thus demonstrating that efflux is a relevant component of the level of fluoroquinolone resistance in these clinical isolates. 

**Figure 3:**
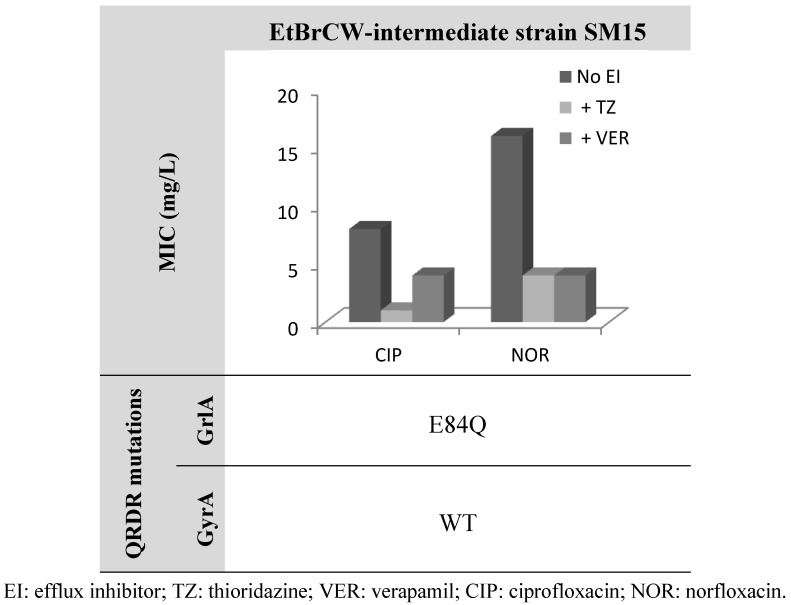
Effect of the efflux inhibitors thioridazine (TZ) and verapamil (VER), at a subinhibitory concentration (12.5 mg/L and 200 mg/L, respectively), on the MIC values of ciprofloxacin and norfloxacin for the EtBrCW-intermediate strain SM15. WT: wild-type sequence (no mutations).

This last observation is further supported by data on strain SM15, classified as EtBrCW-intermediate and showing low level resistance to fluoroquinolones and the single isolate among the EtBrCW-intermediate isolates that carry solely a single mutation in *grlA* QRDR and no mutation in *gyrA* (Figure 3). This strain presented MICs of 8 mg/L for ciprofloxacin and 16 mg/L for norfloxacin, which are near the breakpoint concentrations for an isolate to be considered resistant to these antibiotics (according to CLSI guidelines [33]). These values are in accordance to data in the literature for isolates carrying a single GrlA mutation [32]. However, the addition of efflux inhibitors lead to a reduction of these MIC levels, which, in the particular case of TZ, drop to the susceptibility levels for both ciprofloxacin and norfloxacin (1 mg/L and 4 mg/L, respectively) (Figure 3). 

These results further strengthen the importance of efflux on fluoroquinolone resistance in *S. aureus*, in particular in the early stage of the acquisition of mutations in the QRDR of *grlA* and *gyrA* genes. Strain SM15 may represent an intermediate stage of the emergence of fluoroquinolone resistance, with balanced contributions from both efflux and mutation to the resistance phenotype. 

In fact, recent studies from independent groups, working with different bacterial species, suggest that efflux systems may be a first response of the cell to cope with antimicrobial agents, enabling the cell to survive and acquire other, more stable resistance mechanisms, such as target gene mutations for fluoroquinolones, that will then provide a high-level resistance phenotype, as was recently demonstrated for *Escherichia coli* [34] and *Mycobacterium tuberculosis* [35]. In *S. aureus*, some evidence has also been found for this role of efflux pumps as a first-line defense mechanism towards noxious compounds [36,37] that are supported by data on clinical strains [6,7,8,38].

### 2.3. Contribution of Efflux to Biocide Reduced Susceptibility

Biocides differ greatly from antibiotics in their mechanism of action; whereas antibiotics have precise cell targets, biocides usually act upon several cellular targets [39]. They have an important role in infection control in healthcare settings, where they are currently used in a variety of products that are applied in the washing and disinfection of the environment and medical devices. They are also used as antiseptics for patients and healthcare professionals in hand hygiene, skin disinfection prior to invasive procedures and mucous disinfection [39]. Among these biocides, antiseptic formulations containing chlorhexidine or quaternary ammonium compounds, together with alcohol-based preparations, are the most commonly used for skin disinfection and hand hygiene, respectively [40]. Apart from healthcare settings, these compounds are widespread in industry, being also increasingly employed in the community setting [41]. Concern regarding the emergence of clinical strains showing reduced susceptibility or tolerance to biocides has been increasing in the last decade, with particular focus on the potential role of biocides as a selective force of antibiotic-resistant bacteria [42]. 

Reduced susceptibility to biocides in *S. aureus* is mainly associated with efflux pumps that are encoded in plasmids, including the efflux systems, QacA/B and Smr [5]. Nevertheless, the several chromosomally-encoded multidrug efflux pumps that have been described so far in *S. aureus* also have in their substrate profile a wide variety of biocides. Therefore, it is important to ascertain the contribution of these efflux systems to the biocide “susceptibility” profile in clinical isolates. 

**Figure 4:**
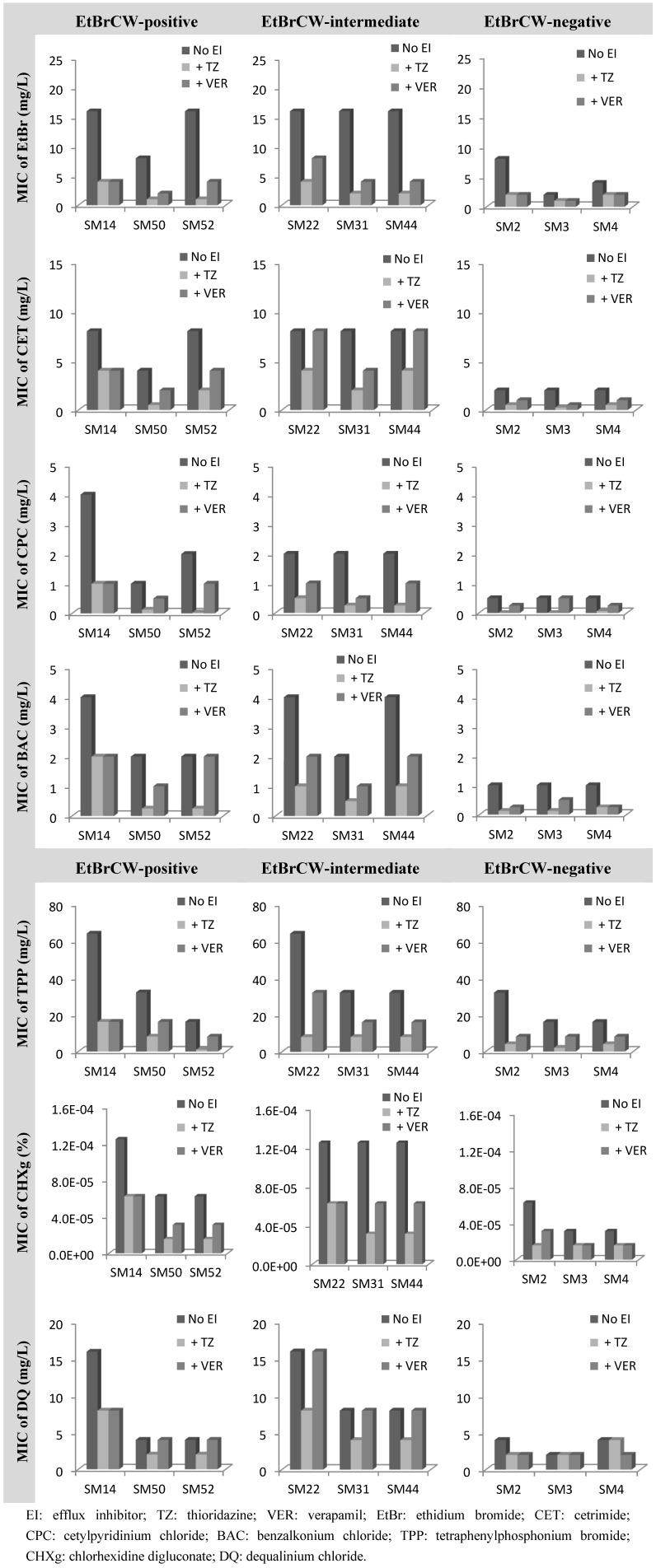
Effect of the efflux inhibitors thioridazine (TZ) and verapamil (VER), at subinhibitory concentrations (12.5 mg/L and 200 mg/L, respectively), on the MIC values of several biocides for representative isolates of the EtBrCW-positive, EtBrCW-intermediate and EtBrCW-negative groups.

The collection of 52 ciprofloxacin-resistant *S. aureus* isolates was also tested for susceptibility to biocides by determination of MICs of several biocides, namely the quaternary ammonium compounds, cetrimide, cetylpyridinium chloride and benzalkonium chloride, the bisbiguanidine chlorhexidine digluconate, pentamidine, tetraphenylphosphonium bromide and dequalinium chloride. MICs of EtBr were also determined, since this compound was used as a marker for efflux activity. Data for representative isolates can be found in Figure 4. Among the compounds tested, it could be observed that the MICs for EtBrCW-positive and -intermediate isolates were generally higher than the ones for EtBrCW-negative isolates. In particular, EtBrCW-positive and -intermediate isolates presented MICs in the following range: EtBr, 8 to 16 mg/L; the quaternary ammonium compounds cetrimide, 4 to 8 mg/L, cetylpyridinium chloride, 1 to 4 mg/L and benzalkonium chloride, 2 to 4 mg/L; tetraphenylphosphonium bromide, 16 to 64 mg/L; chlorhexidine digluconate, 0.00006% to 0.000125%, and dequalinium chloride, 4 to 16 mg/L. The range of MICs for the EtBrCW-negative isolates varied as follows: EtBr, 2 to 8 mg/L; cetrimide, 2 mg/L; cetylpyridinium chloride, 0.5 mg/L; benzalkonium chloride, 1 mg/L, tetraphenylphosphonium bromide, 16 to 32 mg/L, chlorhexidine digluconate, 0.00003% to 0.00006%; and dequalinium chloride, 2 to 4 mg/L (detailed MIC data is provided in Supplementary Table S2, Table S3). No significant difference was found between the groups of isolates for the MICs of pentamidine (data not shown). In sum, the EtBrCW-positive and -intermediate isolates presented MIC values that were two- to eight-fold higher than the ones presented by the EtBrCW-negative isolates (Figure 4). Although this difference is not extensive, it reveals that the efflux-positive and -intermediate isolates can withstand higher concentrations of these biocides. Moreover, it demonstrated a variation of the MICs of biocides according to the efflux capacity of the three groups of isolates (Figure 4). 

To assure that the difference observed in the MIC values of the biocides and EtBr was the result of the higher efflux capacity of the EtBrCW-positive and EtBrCW-intermediate isolates, the effect of TZ and VER on the MICs of these compounds was also evaluated (Figure 4 and Supplementary Table S2, Table S3).

The effect of the efflux inhibitors upon the MIC values of the biocides selected showed that efflux activity has a strong involvement in the reduced susceptibility of *S. aureus* to biocides. For all isolates tested, independently of their efflux capacity, the MICs of all the biocides were reduced two- to 16-fold in the presence of TZ and none to eight-fold (mostly, about two-fold reduction) in the presence of VER, with the highest inhibitory effects observed for the quaternary ammonium compounds and for tetraphenylphosphonium bromide. The biocide for which this effect was lower was dequalinium chloride, with none to two-fold reduction of MICs in the presence of the efflux inhibitors. It could also be observed, in general, that for EtBrCW-positive and -intermediate isolates, the efflux inhibitors could reduce the MICs of the several compounds to levels similar or lower than the ones presented by the EtBrCW-negative isolates. Altogether, these results indicate that efflux activity contributes to reduced susceptibility to biocides in *S. aureus*. 

We have previously referred to the lower capacity of fluoroquinolone MIC reduction by VER, when compared to TZ [7]. Here, we provide experimental data on this difference and show that the same effect is observable for biocides, as well.

Among the EtBrCW-positive isolates, SM52 is the only to carry a plasmid with the gene for the efflux pump Smr [7]. This pump is associated with low-level resistance to biocides and EtBr [43] and is found in a low prevalence (around 10%) in clinical *S. aureus* isolates [44,45,46]. The MIC values presented by the isolate SM52 are equal or lower than the ones of the remaining EtBrCW-positive and -intermediate isolates (that carry no Smr or QacA/B efflux pump), thus showing that the potentially active chromosomal multidrug efflux pumps can confer a resistance level to biocides similar or higher than the Smr plasmid-encoded efflux pump. 

The results described suggest that active efflux systems can be responsible for reduced susceptibility to biocides in *S. aureus*. The concentration in use in which these compounds are applied for washing and disinfection in healthcare settings is higher than the MICs values determined for these isolates. For example, chlorhexidine is used in concentrations that range between 0.2 to 4%, much higher than the highest MIC found in these isolates. This may suggest that this observed reduced susceptibility to biocides is not clinically relevant. Nevertheless, the misuse of these biocide formulations, especially in terms of the application time, together with contaminating residues left after use, may provide opportunities for more tolerant bacteria to be maintained and proliferate in these environments [47,48,49]. Furthermore, concern arises regarding biocide-resistant/tolerant strains and their role in the selection of antibiotic-resistant strains. Studies have related contradictory data concerning the relation between antibiotic and biocide resistance, but much evidence has been gathered that supports the potential co-selection of strains with reduced susceptibility to biocides by antibiotic-resistant bacteria, and *vice versa*. In the particular case of *S. aureus*, studies have shown that exposure to biocides can induce the overexpression of multidrug efflux pumps in both reference and clinical strains, leading to reduced susceptibility to the inducing biocide, as well as to other biocides and to antibiotics [38,50]. These findings strengthen the importance of increasing our knowledge of efflux as a resistance mechanism in *S. aureus*. 

## 3. Experimental

A collection of 52 ciprofloxacin-resistant *S. aureus* isolates was studied [7]. The pan-susceptible reference strain, *S. aureus* ATCC25923, and its ethidium bromide-adapted counterpart, ATCC25923_EtBr_ [50]. were used as controls. All strains were grown in tryptic soy broth (TSB) at 37 °C with shaking or in trypticase soy agar (TSA) (Oxoid Ltd., Basingstoke, UK). The strain ATCC25923_EtBr_ was grown in TSB/TSA supplemented with 50 mg/L of EtBr. Antibiotics were purchased from different sources, as follows: ciprofloxacin (Fluka Chemie GmbH, Buchs, Switzerland) and norfloxacin (ICN Biomedicals Inc., Aurora, OH, USA). EtBr, biocides (benzalkonium chloride, tetraphenylphosphonium bromide, pentamidine isothionate salt, cetylpyridinium chloride, cetrimide, dequalinium chloride and chlorhexidine digluconate) and efflux inhibitors (thioridazine and verapamil) were acquired from Sigma-Aldrich (Madrid, Spain). All efflux inhibitors solutions were prepared in deionized water on the day of the experiment and kept protected from light. 

For the EtBr-agar cartwheel method [12], each culture was swabbed onto TSA plates containing EtBr concentrations ranging from 0.5 to 2.5 mg/L in increments of 0.5 mg/L EtBr. *S. aureus* ATCC25923 and ATCC25923_EtBr_ were used as negative and positive controls for efflux activity, respectively [50]. The plates were incubated at 37 °C during 16 hours, after which the minimum concentration of EtBr associated with the bacterial mass that produced fluorescence under UV light was recorded in a Gel-Doc XR apparatus (Bio-Rad, Hercules, CA, USA). Isolates showing fluorescence at lower EtBr concentrations have potentially less active efflux systems than isolates for which fluorescence is only detected at higher concentrations of EtBr [12]. Thus, isolates were classified according to the emitted fluorescence registered, namely isolates showing emission of fluorescence at 0.5–1 mg/L EtBr were denominated EtBrCW-negative (with no potential active efflux systems); isolates showing emission of fluorescence at 1.5–2.0 mg/L EtBr were denominated EtBrCW-intermediate; and isolates emitting fluorescence only at the maximum concentration of EtBr tested (2.5 mg/L) were denominated EtBrCW-positive (with potential active efflux systems).

Efflux assays by real-time fluorometry were performed in a Rotor-Gene 3000™ thermocycler, together with real-time analysis software (Corbett Research, Sydney, Australia) [13]. Cultures were grown in TSB medium at 37 °C with shaking until an optical density at 600 nm (OD_600_) of 0.6. The cells were collected by centrifugation at 13,000 rpm for 3 minutes and the pellet washed twice with a 1X phosphate buffered saline (PBS) solution. EtBr-loaded cells were prepared by incubating a cellular suspension with an OD_600_ of 0.3 with 0.25, 0.5 and 1 mg/L EtBr for EtBrCW-negative, -intermediate and -positive cultures, respectively, plus 200 mg/L of verapamil (a sub-inhibitory concentration) at 25 °C for 60 minutes. After EtBr accumulation, cells were collected by centrifugation and resuspended in 1X PBS to an OD_600_ of 0.6. Several parallel assays were then run in 0.1 mL final volume corresponding to 0.05 mL of the EtBr loaded cells (final OD_600_ of 0.3) incubated with 0.05 mL of (1) PBS only, (2) glucose 0.8% only (final concentration of 0.4%), (3) 400 mg/L of verapamil only (final concentration of 200 mg/L) and (4) glucose 0.8% plus 400 mg/L of verapamil (final concentrations of 0.4% and 200 mg/L, respectively). Efflux assays were conducted in the Rotor-Gene 3000™ at 37 °C, and the fluorescence of EtBr was measured (530/585 nm) at the end of every cycle of 10 seconds, for a total period of 10 minutes. The raw data obtained was then normalized against data obtained from non-effluxing cells (cells from the control tube with only 200 mg/L VER), at each point, considering that these correspond to the maximum fluorescence values that can be obtained during the assay. The relative fluorescence thus corresponds to the ratio of fluorescence that remains per unit of time, relatively to the EtBr-loaded cells.

For determination of minimum inhibitory concentrations (MICs), cultures were grown in Mueller-Hinton broth (MH, Oxoid) at 37 °C. MICs for antibiotics were determined by the two-fold broth microdilution method and evaluated according to the CLSI breakpoints [33]. MICs for EtBr and biocides were also determined using the two-fold broth microdilution method. After an 18 hour incubation period at 37 °C, the MIC values were recorded, corresponding to the lowest concentration of antimicrobial compound that presented no visible growth. To evaluate the effect of efflux inhibitors on the MIC values, parallel cultures were tested in media containing varying concentrations of the antimicrobial compound in the absence and presence of the efflux inhibitors thioridazine and verapamil at the sub-inhibitory concentrations of 12.5 mg/L and 200 mg/L and equivalent bacterial inoculums. The cultures were incubated for 18 hours and growth evaluated visually. All assays were determined in triplicate.

For the screening of mutations conferring fluoroquinolone resistance, internal fragments comprising the QRDR of *grlA* and *gyrA* genes were amplified using primers previously described [7]. The reaction mixture (50 μL) contained 2.5 U of Taq Polymerase (Fermentas Inc., Ontario, Canada), 1X Taq buffer (Fermentas), 25 pmol of each primer, 0.2 mM of dNTP and 1.75 mM of MgCl_2_. The PCR reactions were conducted in a thermocycler Mastercycler personal 5332 (Eppendorf AG, Hamburg, Germany). The amplification conditions were as follows: DNA was denatured at 94 °C for 4 minutes, followed by 35 cycles of denaturation at 94 °C for 30 seconds, annealing at 50 °C for 30 seconds and extension at 72 °C for 1 minute, followed by a step of final extension at 72 °C for 5 minutes. Amplification products were purified and sequenced in both strands using the same set of primers. Sequences were analyzed and aligned using the freeware programs, BioEdit and ClustalW, respectively. 

## 4. Conclusions

The results described demonstrate that the two EtBr-based approaches used, namely the EtBrCW method and real-time fluorometry, are valuable techniques to screen and characterize efflux activity in clinical isolates of *S. aureus*. These isolates were classified according to their capacity to efflux EtBr and the MIC determination in the presence of efflux inhibitors allowed the correlation of this efflux activity with resistance to fluoroquinolones and some biocides, including quaternary ammonium compounds and chlorhexidine, antimicrobials widely used in healthcare settings. These results show that EtBr is indeed a good screening marker for efflux activity leading to resistance to fluoroquinolones and biocides.

The data compiled in this study, together with results from previous studies from our group and other colleagues, indicate that efflux is a major mechanism in the first stage of development of resistance to antimicrobial compounds, in this case, fluoroquinolones. That is, activation of efflux systems by fluoroquinolones could promote *S. aureus* survival in “stress conditions”, allowing the bacteria to acquire and accumulate target gene mutations that are associated with high-level resistance. Furthermore, the demonstration that this same efflux activity can sustain higher tolerance of *S. aureus* cells to clinically relevant antiseptics and disinfectants, which could also, in turn, potentiate antibiotic resistance, strengthen the importance of this long neglected resistance mechanism to the persistence and proliferation of antibiotic/biocide-resistant *S. aureus* in the hospital environment.

## Supplementary Files

Supplementary File 1
