# Supplementary material for: Resistance to Antimicrobials Mediated by Efflux Pumps in Staphylococcus aureus
Source: Antibiotics (Basel). 2013 Mar 13;2(1):83–99. doi: 10.3390/antibiotics2010083 (PMC4790300; doi:10.3390/antibiotics2010083)
Supplement: Supplementary File 1 [file antibiotics-02-00083-s001.pdf]

Supplementary Material for

Resistance to Antimicrobials Mediated by Efflux Pumps in *Staphylococcus aureus*

Table S1. MIC values of ciprofloxacin and norfloxacin for strains representative of the EtBrCW-positive, EtBrCW-intermediate and EtBrCW-negative groups, in the absence and presence of subinhibitory concentrations of the efflux inhibitors thioridazine and verapamil.

Strain	MIC (mg/L)					
	CIP			NOR		
	No EI	+ TZ	+ VER	No EI	+ TZ	+ VER
EtBrCW-positive						
**SM14**	256	32	128	1024	128	256
**SM50**	64	16	16	256	32	64
**SM52**	16	8	8	64	32	64
EtBrCW-intermediate						
**SM15**	8	1	4	16	4	4
**SM22**	128	16	64	512	128	256
**SM31**	64	32	64	256	128	128
**SM44**	256	32	128	512	64	128
EtBrCW-negative						
**SM2**	32	16	16	128	32	64
**SM3**	16	8	8	64	32	64
**SM4**	8	8	8	64	32	64

CIP: ciprofloxacin; NOR: norfloxacin; EI: efflux inhibitor; TZ: thioridazine; VER: verapamil. Thioridazine and verapamil were used at 12.5 mg/L and 200 mg/L, respectively.

Table S2. MIC values of ethidium bromide for strains representative of the EtBrCW-positive, EtBrCW-intermediate and EtBrCW-negative groups, in the absence and presence of subinhibitory concentrations of the efflux inhibitors thioridazine and verapamil.

Strain	EtBr MIC (mg/L)		
	No EI	+ TZ	+ VER
EtBrCW-positive			
**SM14**	16	4	4
**SM50**	8	1	2
**SM52**	16	1	4
EtBrCW-intermediate			
**SM22**	16	4	8
**SM31**	16	2	4
**SM44**	16	2	4
EtBrCW-negative			
**SM2**	8	2	2
**SM3**	2	1	1
**SM4**	4	2	2

EtBr: ethidium bromide; EI: efflux inhibitor; TZ: thioridazine; VER: verapamil. Thioridazine and verapamil were used at 12.5 mg/L and 200 mg/L, respectively.

Table S3. MIC values of biocides for strains representative of the EtBrCW-positive, EtBrCW-intermediate and EtBrCW-negative groups, in the absence and presence of subinhibitory concentrations of the efflux inhibitors thioridazine and verapamil.

	**MIC (mg/L)**															**MIC (%)**		
	**CET**			**CPC**			**BAC**			**TPP**			**DQ**			**CHXg**		
**Strain**	**No EI**	**+ TZ**	**+ VER**	**No EI**	**+ TZ**	**+ VER**	**No EI**	**+ TZ**	**+ VER**	**No EI**	**+ TZ**	**+ VER**	**No EI**	**+ TZ**	**+ VER**	**No EI**	**+ TZ**	**+ VER**
EtBrCW-positive																		
**SM14**	8	4	4	4	1	1	4	2	2	64	16	16	16	8	8	0.000125	0.00006	0.00006
**SM50**	4	0.5	2	1	0.125	0.5	2	0.25	1	32	8	16	4	2	4	0.00006	0.000015	0.00003
**SM52**	8	2	4	2	0.03	1	2	0.25	2	16	1	8	4	2	4	0.00006	0.000015	0.00003
EtBrCW-intermediate																		
**SM22**	8	4	8	2	0.5	1	4	1	2	64	8	32	16	8	16	0.000125	0.00006	0.00006
**SM31**	8	2	4	2	0.25	0.5	2	0.5	1	32	8	16	8	4	8	0.000125	0.00003	0.00006
**SM44**	8	4	8	2	0.25	1	4	1	2	32	8	16	8	4	8	0.000125	0.00003	0.00006
EtBrCW-negative																		
**SM2**	2	0.5	1	0.5	0.03	0.25	1	0.125	0.25	32	4	8	4	2	2	0.00006	0.000015	0.00003
**SM3**	2	0.25	0.5	0.5	0.03	0.5	1	0.125	0.5	16	2	8	2	2	2	0.00003	0.000015	0.000015
**SM4**	2	0.5	1	0.5	0.06	0.25	1	0.25	0.25	16	4	8	4	4	2	0.00003	0.000015	0.000015

CET: cetrimide; CPC: cetylpyridinium chloride; BAC: benzalkonium chloride; TPP: tetraphenylphosphonium bromide; DQ: dequalinium chloride; CHXg: chlorhexidine digluconate; EI: efflux inhibitor; TZ: thioridazine; VER: verapamil. Thioridazine and verapamil were used at 12.5 mg/L and 200 mg/L, respectively.
